# Classification of Stereo-EEG Contacts in White Matter vs. Gray Matter Using Recorded Activity

**DOI:** 10.3389/fneur.2020.605696

**Published:** 2021-01-06

**Authors:** Patrick Greene, Adam Li, Jorge González-Martínez, Sridevi V. Sarma

**Affiliations:** ^1^Neuromedical Control Systems Lab, Institute for Computational Medicine, Biomedical Engineering, Johns Hopkins University, Baltimore, MD, United States; ^2^Neurosurgery, Cleveland Clinic, Cleveland, OH, United States

**Keywords:** stereo-electroencephalography, SEEG, white matter, classification, power spectrum, bipolar reference

## Abstract

For epileptic patients requiring resective surgery, a modality called stereo-electroencephalography (SEEG) may be used to monitor the patient's brain signals to help identify epileptogenic regions that generate and propagate seizures. SEEG involves the insertion of multiple depth electrodes into the patient's brain, each with 10 or more recording contacts along its length. However, a significant fraction (≈ 30% or more) of the contacts typically reside in white matter or other areas of the brain which can not be epileptogenic themselves. Thus, an important step in the analysis of SEEG recordings is distinguishing between electrode contacts which reside in gray matter vs. those that do not. MRI images overlaid with CT scans are currently used for this task, but they take significant amounts of time to manually annotate, and even then it may be difficult to determine the status of some contacts. In this paper we present a fast, automated method for classifying contacts in gray vs. white matter based only on the recorded signal and relative contact depth. We observe that bipolar referenced contacts in white matter have less power in all frequencies below 150 Hz than contacts in gray matter, which we use in a Bayesian classifier to attain an average area under the receiver operating characteristic curve of 0.85 ± 0.079 (SD) across 29 patients. Because our method gives a probability for each contact rather than a hard labeling, and uses a feature of the recorded signal that has direct clinical relevance, it can be useful to supplement decision-making on difficult to classify contacts or as a rapid, first-pass filter when choosing subsets of contacts from which to save recordings.

## 1. Introduction

Over 15 million epilepsy patients worldwide and 1 million in the US suffer from drug-resistant epilepsy (1–4). Approximately 50% of such patients have focal drug-resistant epilepsy, where a specific region or set of regions in the brain is the source of the abnormal electrical activity resulting in seizures. This region, termed the epileptogenic zone (EZ), is the area of cortex that is necessary and sufficient for initiating seizures and whose removal or disconnection is necessary for complete abolition of seizures (5–8). When successful, surgical resection treatments stop seizures or allow them to be controlled with medications. Outcomes depend critically on the clinician's ability to accurately identify the EZ.

In cases where standard methods such as EEG are inconclusive in determining the EZ, a more invasive modality called stereo-electroencephalography (SEEG) may be used. With SEEG, multiple depth electrodes are inserted into a patient's brain, each with 10 or more contacts along its length. This allows relatively high resolution mapping of the electrical activity in both shallow and deep structures of the brain. One drawback however is that a significant fraction of the contacts will reside in white matter or other areas of the brain which can not be epileptogenic themselves (although they can contribute to propagation of seizures). Thus, accurate localization of the EZ begins with determining which electrode contacts provide useful information about brain activity. Generally these are contacts which reside in gray matter or close to it. Especially in cases where patients have a large number of electrodes implanted, it is highly convenient for the SEEG reader to have such contacts readily identified. If a contact in gray matter is ignored because it is incorrectly believed to be in white matter, a part of the EZ may be missed. On the other hand, if a contact in white matter is assumed to be in gray matter, it may confound localization. Further, in studies that use network-based analysis to assist in identification of the epileptogenic zone, it is important to have a complete labeling of all contacts with information about which are likely to be in gray vs. white matter (9–12).

Another aspect of presurgical planning is functional mapping of the patient's brain to determine the borders of highly important areas such as those used for speech or movement which should be avoided as much as possible during surgery. Functional mapping involves sub-threshold stimulation of both white matter and gray matter regions of the patient's brain and observing the electrical and behavioral response. Stimulation parameters in white matter must be tuned differently than those for gray matter, and the results of the stimulation are interpreted differently as well. An incorrect label of a contact as being white matter will result in the wrong stimulation settings and potentially errors in functional mapping.

Many epilepsy treatment centers, including those not invested in research and data analysis such as the aforementioned network analysis methods, have resource and time constraints in both the presurgical planning and functional mapping stages which do not allow gray/white matter labeling of all recording contacts. Instead, labels are identified for only a fraction of contacts around the likely EZ or perhaps even for no contacts at all. The latter approach, while taking little time, relies heavily on physician experience and is potentially prone to errors in localization. For those contacts which are labeled however, whether this is a full or partial labeling, the standard approach uses co-registration of MRIs with CT scans, which are overlaid onto brain atlases to anatomically identify regions including white matter (13). Contacts are then classified based on majority-voting or distance measures using white matter voxel masks derived from segmentations of the T1 MRI (e.g., FreeSurfer) (14, 15). However, MRI-based methods, including those that combine other modalities like diffusion tensor imaging, take significant amounts of time to analyze and annotate, hence the tendency toward partial labeling in many centers, and accuracy depends highly on the quality of images (13, 16, 17). While software exists to assist with and automate parts of the segmentation and gray/white matter classification process, to our knowledge all such software is based on MRI and CT images (15). A method which requires minimal time and uses non-imaging data would give clinicians an additional source of information that may be helpful, especially at centers where labeling is currently not done at all or in cases of contacts whose position is uncertain.

In this paper we present a fast, automated Bayesian method for estimating the probability that an electrode contact is in white matter based only on the spectral content of the recorded signal and the relative contact depth. We tested our method using SEEG recordings from 29 drug resistant epilepsy patients who underwent invasive monitoring for treatment purposes. We found that our classifier achieved an average area under the receiver operating characteristic curve of 0.845 ± 0.079 (SD) across patients. Our method is accurate enough that it can be used to supplement MRI-based approaches to further improve accuracy and reduce the time needed to find an informative subset of recording contacts. In particular, for treatment centers where electrode contact labeling is not done due to time or resource limitations, or is done for only a small subset of contacts, our method can be thought of as identifying contacts which have strong, lower correlation signals relative to their neighbors—typically gray matter contacts—without requiring any additional work or scans on the part of the clinician and very little additional time. As one step within a larger automation pipeline for aiding identification of the EZ, we believe that such a method may prove useful in increasing speed and localization accuracy regardless of whether the center uses a full, partial, or no labeling.

## 2. Materials and Methods

### 2.1. Data Collection

**Figure 1:**
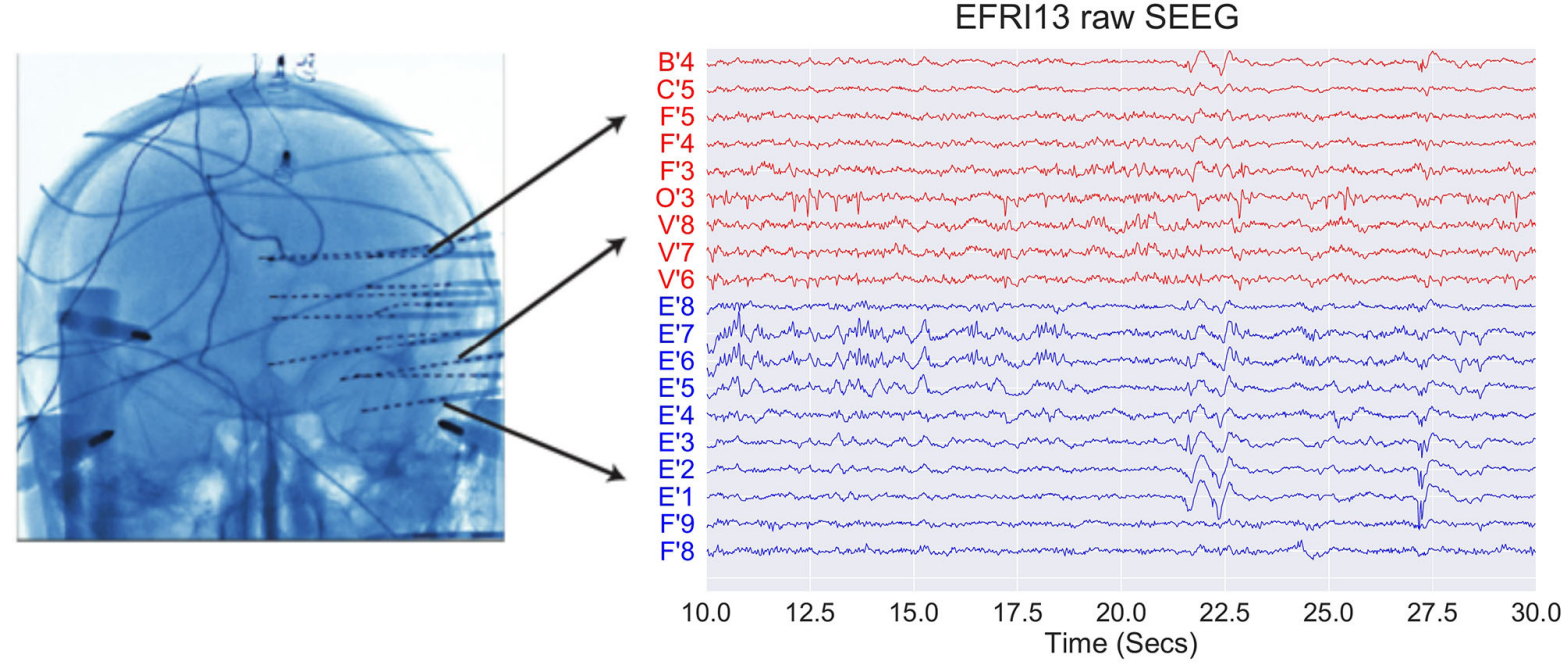
Example of SEEG electrode placement and raw voltage data for sample subject EFRI13 over a 20 s snapshot. Blue traces denote gray matter and red traces denote white matter. Each trace has a scale of 400 uV and has a monopolar reference.

EEG data from 29 epilepsy patients who underwent intracranial EEG monitoring, which included depth electrodes with stereotactic EEG (SEEG) were selected from the Cleveland Clinic. Patients exhibiting the following criteria were excluded: patients with no seizures recorded, pregnant patients, patients less than 5 years of age, and patients with an EEG sampling rate less than 1000 Hz. For each patient, we aggregated interictal data where the patient was not seizing. Figure 1 shows an example SEEG implantation with common reference voltage data from white and gray matter contacts.

Data were recorded using a Nihon Kohden (Tokyo, Japan) acquisition system with a typical sampling rate of 1,000–2,000 Hz. Signals were referenced to a separate, common electrode placed subcutaneously on the scalp. The clinicians then clipped snapshots of SEEG data and passed it through a secure transfer for analysis in the form of the European Data Format (EDF) (18). We discarded electrodes from further analysis if they were deemed excessively noisy by clinicians, or were not relevant (for example: reference, or EKG, or not attached to the brain). We stored data in the BIDS-iEEG format and performed processing using Python 3.6, MNE-Python, and MNE-BIDS, as well as MATLAB (19–23). Figures were generated using MATLAB and Matplotlib (24). An implementation of our classifier is available at https://github.com/Patrick-Greene/WM-classifier.

Decisions regarding the need for invasive monitoring and the placement of electrode arrays were made independently of this work and part of routine clinical care. All data were acquired with approval of the local Institutional Review Board (IRB) at each clinical institution. The acquisition of data for research purposes was completed with no impact on the clinical objectives of the patient stay. Digitized data were stored in an IRB-approved database compliant with Health Insurance Portability and Accountability Act (HIPAA) regulations.

### 2.2. Feature Extraction

For each electrode contact, the raw, common reference SEEG data were bipolar referenced by subtracting off the signal from the adjacent contact closer to the tip of the shank (i.e., the next deeper or more mesial electrode). The contact at the end of the shank had the previous contact subtracted. White matter contacts were those identified by the treating physician as being in white matter using the standard MRI and CT coregistration procedure described previously. Ambiguous electrodes were labeled according to which class the clinician felt was most correct (i.e., if an electrode appeared to be more in gray matter than white matter, it would be labeled as gray matter), and this was the “ground truth” used in our training set and for computing test set error rates. For the purposes of this study, we simplified the analysis by removing all contacts which were not in either white or gray matter, for example contacts in ventricles or outside the cortical surface, during preprocessing.

**Figure 2:**
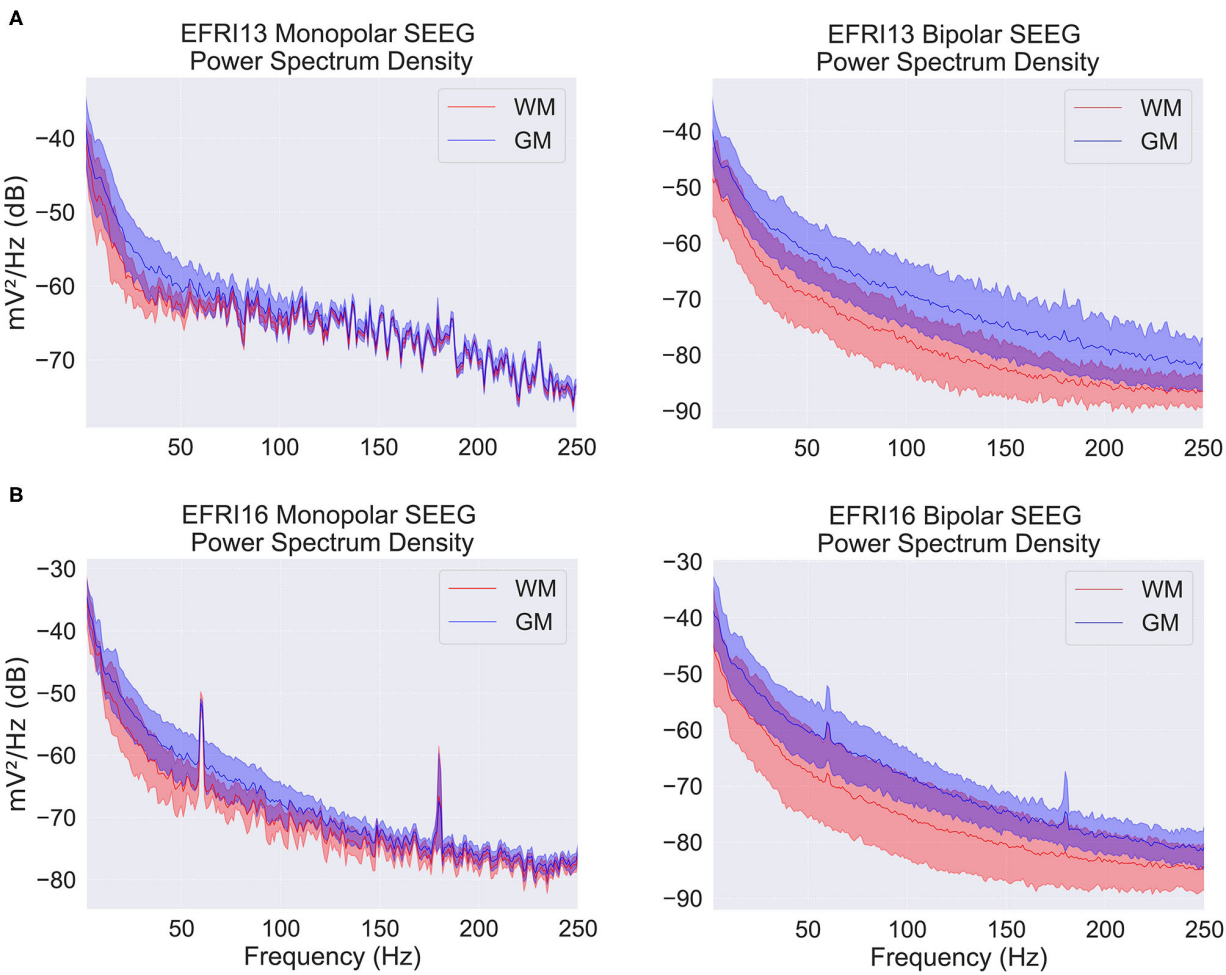
Power spectral density (PSD) plots for two example patients over a snapshot of 10 s with monopolar and bipolar referencing. EFRI13 **(A)**, and EFRI16 **(B)** are shown with PSD segregated based on contacts in white matter (red traces) or gray matter (blue traces). Shaded regions denote one standard deviation. Spikes at multiples of 60 Hz are due to line frequency noise.

We chose two features for each electrode contact which preliminary exploration and conversations with clinicians identified as potentially useful in classification. Feature one was the average vertical shift in the contact's power spectrum (in log scale) relative to the average power spectrum over all contacts within that patient. This was computed as the average difference across all frequencies from 1 to 150 Hz between each contact's power spectral density and the average power spectral density. This feature takes into account the fact that white matter contacts tend to have both smaller and more correlated signals because they are further from the neural source of the signal, the gray matter. Figure 2 shows examples of the average power spectrum for white and gray matter contacts under both common and bipolar referencing schemes. The lower overall power in white matter contacts can clearly be seen, particularly in the bipolar referenced data. We subtract off the average power spectral density across contacts within each patient in order to account for across-patient differences in mean power.

Between 1 and 150 Hz the difference between the gray and white matter power spectra in log scale can be closely approximated by a simple downward shift, allowing us to reduce the relevant information in the spectrum to a single dimension—the size of the shift relative to the average spectrum. For significantly higher frequencies (250+ Hz), both spectra have almost equally low power and thus do not contribute much to classification while also violating the simple shift described above, requiring a higher dimensional feature space. For smaller frequency ranges, the estimate of the shift amount is slightly less accurate because it is averaged over fewer frequencies. We thus use roughly the largest frequency range in which the simple shift approximation still holds. The exact cutoff at 150 Hz is not important though; one can vary the upper frequency limit by 50 Hz in either direction with minimal change in accuracy. The power spectral density was estimated using Welch's method in 10 equally spaced 10 s windows, then averaged across the windows. The spectrum in a 4 Hz band around 60 and 120 Hz was omitted due to line noise. The use of multiple windows helped to average out temporary changes in the spectrum due to movement or other artifacts. The number and length of windows was chosen to balance running time and accuracy of the estimated spectrum. Longer or more windows did not significantly change the estimated feature values.

The second feature for each contact was the contact's distance from the most peripheral electrode shank, where the distance between contacts was normalized to 1. We will refer to this as the contact depth or distance along the shank. The most peripheral contact was defined as the first contact that was not outside the brain. For example, if the first 3 contacts on a shank were outside the brain, contact 4 would have a depth value of 0, contact 5 would have a value of 1, and so forth. The normalization was simply for convenience because all our patients had the same distance between contacts; if applying the method on a heterogeneous set of electrodes, one would use the actual distance along the shank instead. This feature takes into account the spatial distribution of white matter along the electrode.

### 2.3. Bayesian Classification

Our overall goals is, for each contact on an electrode in the test set, to classify it as either being in white or gray matter. We approach this classification problem from a Bayesian perspective, which allows us to give class probabilities for each contact that explicitly take into account its feature values, the overall structure of the brain, and uncertainty in our parameter estimates.

**Figure 3:**
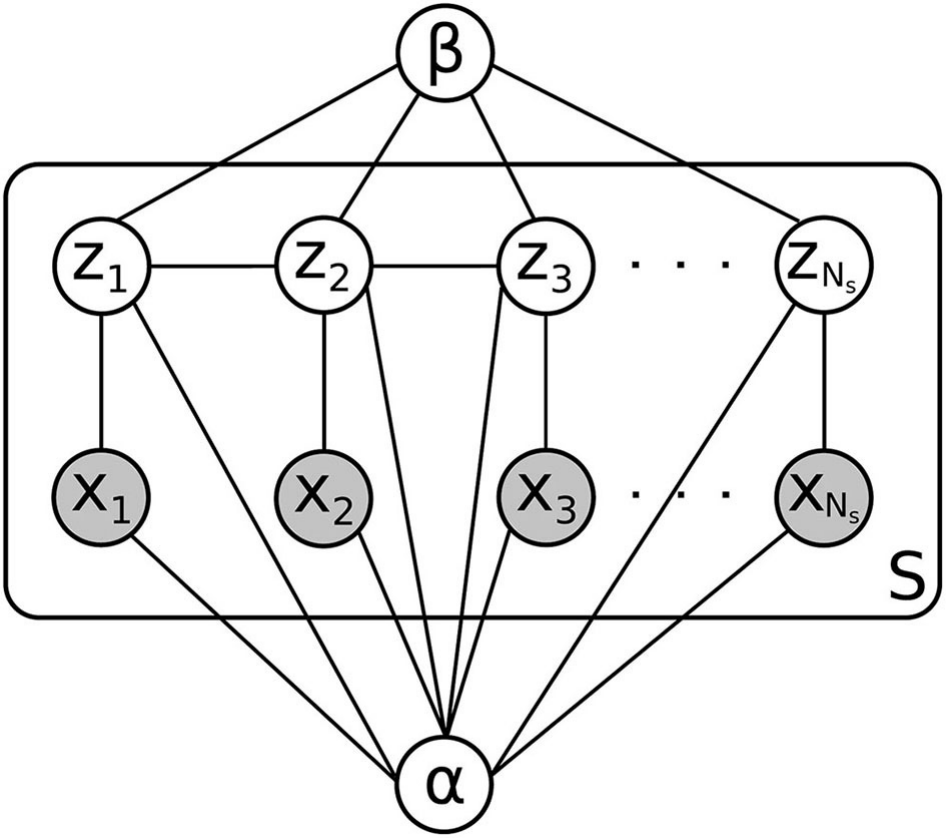
Graphical model for a patient in the test set. There are *S* electrode shanks to be classified, each with *N*_*s*_ electrode contacts. The contact label is given by *z*_*i*_, and the observed features are given by *x*_*i*_. α and β are parameters whose distribution is estimated using the training set *D*, which is not shown here.

With the features described above, we use a kernel density estimator to estimate the continuous feature distributions for both white and gray matter on the training data *D*. The value of the density at any point is given by a distance-weighted average of feature values at nearby training points, where the weighting function is a gaussian *K*_***α***_ centered at the evaluation point and parameterized by the vector of kernel widths ***α***_*wm*_ = (α_*wm*,1_, α_*wm*,2_) or ***α***_*gm*_ = (α_*gm*,1_, α_*gm*,2_) for the white and gray matter distributions, respectively. For a new contact in the test set, we use these distributions to evaluate the probability of observing the contact's feature values, given that it is either in white matter or gray matter (the *likelihood* of the contact's data). This likelihood is calculated for each contact on an electrode shank and, under the assumption that each contact's data is independent conditioned on the class assignment (gray or white matter), the likelihood of the electrode shank as a whole is computed by multiplying together the individual contact probabilities. This independence assumption, along with several others used in the derivation of our classifier, is shown graphically in Figure 3.

We now wish to take into account the large-scale distribution of white and gray matter in the brain. Given the physical structure of the brain, we would find it unlikely for an electrode to have contacts that alternate between white matter and gray matter in rapid succession. More typical would be to have several gray matter contacts in a row, followed by several white matter contacts in a row, perhaps followed by another chunk of gray matter contacts. This tendency for neighboring contacts to have the same classification is captured by our *prior* distribution on labelings, which consists of an exponentially increasing function of a sum of pairwise products between neighboring contact classes. Since the classes are denoted −1 for gray matter and 1 for white matter, neighboring contacts that have the same class assignment increase the prior probability, while contacts that have different assignments decrease the probability, resulting in a tendency toward fewer gray/white matter transitions on each electrode. This prior is a one-dimensional version of an Ising-type prior used for example in image de-noising, where neighboring pixels in an image are assumed more likely to have the same color (25). The degree to which gray/white matter transitions are penalized is determined by a parameter β. For a fixed choice of parameters ***α*** and β, the product of this prior distribution with the likelihood gives, after normalization, the *posterior* probability of a particular labeling of all the contacts on an electrode shank.

We now turn to the problem of determining our unknown parameters. We allow the user to explicitly choose the kernel width ***α***_2_ = (α_*wm*,2_, α_*gm*,2_) in the second feature dimension corresponding to the contact depth. This is because the appropriate kernel width depends on the physical distance between contacts relative to the size of white matter or gray matter structures in the brain. Larger kernel widths mean that the spatial distribution of white or gray matter varies slowly from contact to contact, while smaller widths allow for more rapid variation. For example, if it is known that contacts are spaced several cm apart, the spatial distribution of white or gray matter varies relatively rapidly with respect to this distance, and hence a smaller kernel width would be called for. In our patients, the contacts are 5 mm apart, so we expect the spatial distribution of white or gray matter to be fairly similar between neighboring contacts. We thus use a larger kernel width equal to the spacing between contacts.

Our data set contains only regularly-spaced electrodes, however, electrodes with irregular spacing can also be accommodated by our method. Rather than using normalized units for the contact positions along the shank (feature two), one would use the actual positions in mm. The estimated white and gray matter densities would automatically scale. The limits of the integral used to compute the likelihood (see Supplementary Equation 5) should be modified accordingly, going from halfway between the previous and next contact positions. The ***α***_2_ kernel width can be chosen according to the smallest set of spacings on the electrode. Cross validation on the training set could also be used to choose ***α***_2_ if a more hands-off approach is desired.

Similarly to the classification problem itself, for the remaining parameters ***α***_1_ and β we estimate their posterior distribution in a Bayesian way by calculating the likelihood of the data given the parameters and multiplying it by a prior distribution. In this case we use a diffuse exponential distribution because we do not have any prior knowledge about parameter values.

Finally, we integrate the posterior probability distribution for each electrode shank's labeling with respect to the posterior distribution of the parameters to form the *posterior predictive* distribution. This takes into account uncertainty in our parameter estimates by forming a weighted average of the class probabilities for various possible parameter values, with weights determined by how probable the parameter values are given the training data. In practice, we estimate this integral by using a truncated gaussian approximation to the posterior parameter distribution (truncated because the parameters must be positive), and average the posterior class probability with respect to samples drawn from this distribution. In the results shown, we average across 100 samples. To compute the probability that any individual contact is in white matter, we sum the posterior predictive over all possible labelings of the other contacts. A detailed mathematical exposition of our method can be found in the Supplementary Material.

### 2.4. Training and Testing

We primarily used leave-one-out cross validation to train and test our method, although we also report results for four-fold cross validation. For a given test patient, the remaining 28 patients were used as training data to estimate the feature distributions for both the white and gray matter classes. We measure overall accuracy by calculating a receiver operating characteristic (ROC) curve and measuring the area under the curve (AUC). A point on the ROC curve is found by picking a probability threshold between 0 and 1, then classifying all contacts on all electrode shanks by whether their estimated probability of being in white matter falls above or below the probability threshold. Contacts with estimated probability above the threshold are classified as being in white matter, and those below the threshold are classified as being in gray matter. The resulting true positive rate—the number of correctly identified white matter contacts divided by the total number of true white matter contacts, and the false positive rate—the number of contacts identified as white matter which were actually in gray matter, divided by the total number of true gray matter contacts, is then calculated. Plotting the true positive rate vs. the false positive rate for various values of the threshold probability produces the ROC curve. Higher threshold probabilities result in fewer false positives but also fewer true positives, and vice versa for lower thresholds, so that ROC curves increase from (0, 0) to (1, 1) as the threshold is lowered. The area under an ROC curve is a summary measure of how efficiently the classifier trades off false positives for true positives, with a maximum value of 1.

## 3. Results

**Figure 4:**
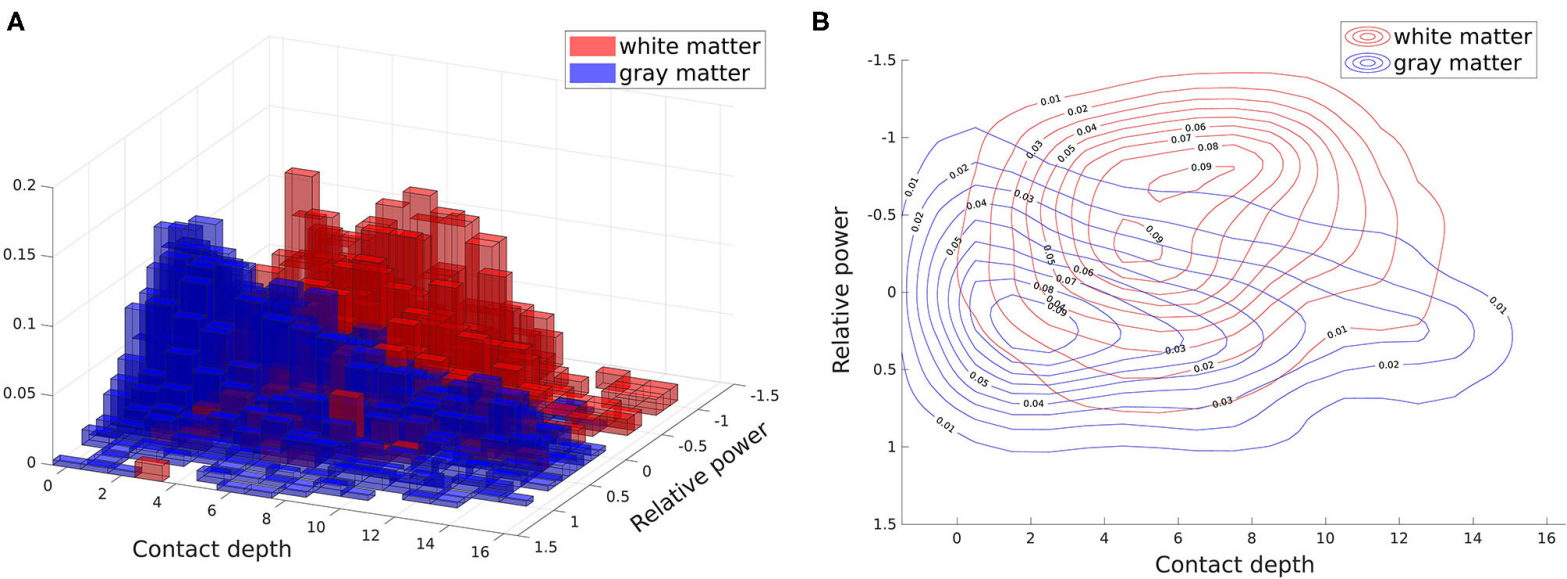
Two dimensional white and gray matter distributions of features: power spectrum difference from average (relative power) and contact depth. These distributions (specifically the training subsets of them) form our white and gray matter likelihoods. All contacts from all patients are shown here, with the feature distribution of white matter contacts shown in red and the distribution for gray matter contacts shown in blue. **(A)** Histogram. **(B)** Overlaid contour plots of estimated kernel densities.

The normalized histograms of the white and gray matter training data are shown together in Figure 4A. We then use kernel density estimators as described previously to give smoothed estimates of each of these distributions. Overlaid contour plots of the density estimates are shown in Figure 4B. In estimating the posterior parameter distributions, we found the average mean of the α_1_ kernel width posterior distributions to be 0.135 for gray matter and 0.156 for white matter (averaged across patients), and the average standard deviations of these posterior distributions to be 0.012 and 0.015, respectively (averaged across patients). The larger kernel width for the white matter distribution is expected given that there are fewer contacts in white matter than gray matter, resulting in fewer data points to estimate the feature distribution. For β, the average mean of its posterior distribution was 7.738 across patients, with an average standard deviation of its distribution of 0.249. The small standard deviations of the posterior distributions relative to their means indicates that the parameters are well-estimated with a 28 patient training set.

**Figure 5:**
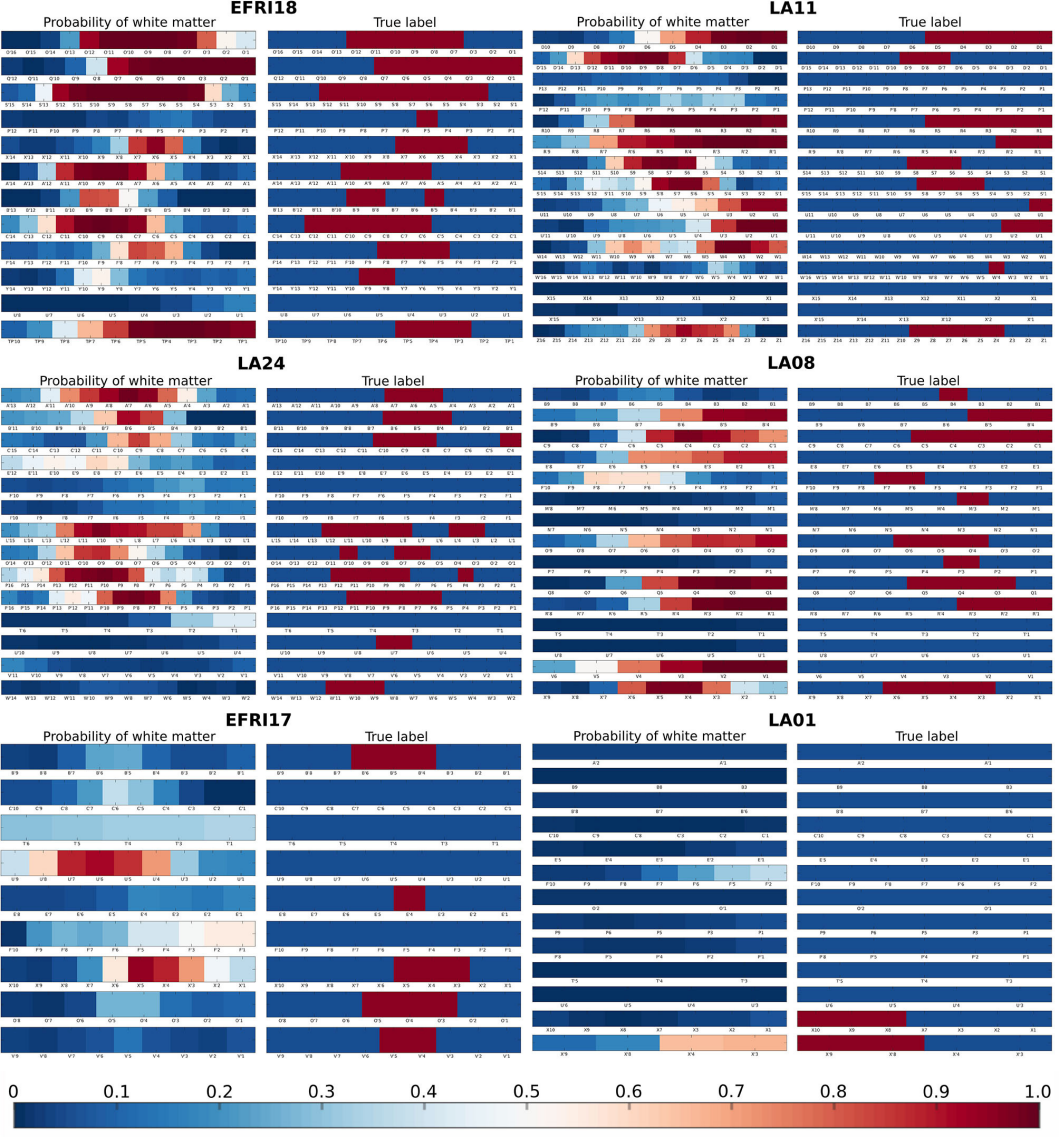
Examples of estimated white matter probabilities for several patients. For each patient, the left column is the probabilities estimated by our classifier, while the right column is the reference labeling done by clinicians using MRI+CT. Each row represents one electrode shank, with individual contacts labeled below. In the reference labeling, red indicates that the contact was labeled as white matter, blue indicates gray matter. In the estimated probabilities, red indicates higher probability of a contact being in white matter according to our classifier. First row: Two of the best patients (EFRI18, LA11), each with > 0.9 AUC. Second row: Two median patients (LA24, LA08), each with 0.86 AUC. Third row: The two worst patients (EFRI17, LA01), representing 0.65 and 0.62 AUC, respectively.

Figure 5 shows example outputs for six patients: the two patients with highest overall accuracy, two patients at the median level of accuracy, and the two least accurate patients. For the high accuracy patients, we see that nearly all gray and white matter contacts are correctly identified with high confidence - probability near 1 for true white matter contacts and probability near 0 for true gray matter contacts. For the median accuracy patients we see some mislabelings, particularly near the transitions between white and gray matter. In patient LA24 a section of white matter spanning three contacts is missed completely, and in patient LA08 sections of gray matter on two electrodes are indicated as likely to be in white matter. Labelings are also done with lower confidence, shown by the lighter reds and blues, particularly in LA24, which denote probabilities closer to 0.5. However, the estimated probabilities still do a good job of tracking the white matter distribution overall. In the patient with the second worst AUC, EFRI17, most of the white matter contacts are missed and a spurious white matter region is found on one of the electrodes. Within this spurious region, the U'5, U'4, and U'3 contacts were labeled by clinicians as being in Heschl's gyrus, which is used for acoustic processing and has a high density of white matter tracts through its center (26). To the classifier's credit, most of the missed contacts are indicated with higher white matter probability than surrounding contacts, albeit still below 0.5. In LA01, the patient with the lowest AUC, we see that this poor accuracy is due almost completely to a low true positive rate. There are only five white matter contacts in this patient, and our classifier fails to find any of them. Nearly all the gray matter contacts are correctly identified however. The two worst patients are somewhat outliers in that the third worst patient has an AUC of 0.74, nearly 10 percentage points better. Only 5 patients had an AUC under 0.8, meaning that the median patients with 0.86 AUC are well representative of most patients.

**Figure 6:**
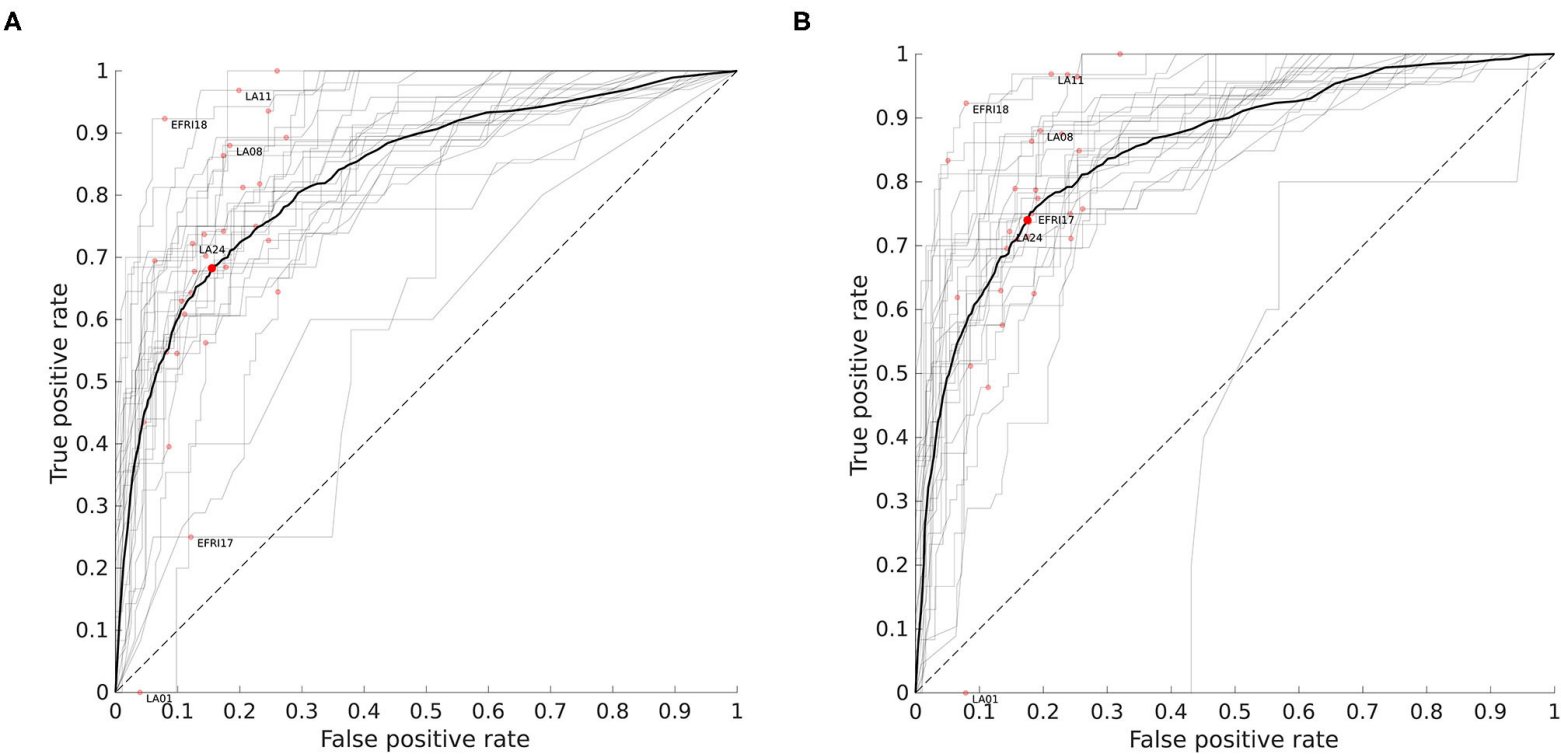
Receiver operating characteristic (ROC) curves for all patients. For a given threshold value *p*_*thresh*_, a contact is classified as white matter if the probability computed by the classifier is above that threshold. Each curve shows how the fraction of false positives (gray matter contacts incorrectly classified as white matter) and true positives (white matter contacts correctly identified) varies with the threshold level. The red dots indicate the point on the curve obtained when *p*_*thresh*_ = 0.5. The area under the curve (AUC) is the integral of the ROC curve and measures overall performance. **(A)** ROC curves when prior smoothing parameter β is automatically integrated over with respect to its estimated posterior distribution (see section 2). Mean AUC = 0.845 ± 0.079 (SD). Patients shown in Figure 5 are labeled. The mean ROC curve is shown in bold. **(B)** ROC curves when β is chosen to maximize AUC over the training set. Mean AUC = 0.859 ± 0.097 (SD).

Figure 6 shows the ROC curves for all 29 patients using two different approaches to setting the prior smoothing parameter β. When we integrate over the posterior distribution of β given the training set as described in the section 2, we obtain the set of ROC curves in Figure 6A. The mean AUC in this case was 0.845±0.079 (SD). If instead we choose a particular value of β for each patient by explicitly maximizing the AUC over the training set, we obtain the set of curves in Figure 6B. Although choosing the AUC-maximizing β predictably results in a slightly higher average AUC (0.859±0.097), the difference is quite small and choosing β in this way is much slower, as it requires running the classifier on the entire training set for multiple values of β. The AUC-maximizing β is approximately 3 on average, while the mean of the posterior distribution of β in our usual approach is 7.7 on average as stated above. While this higher value reduces overall AUC slightly, it also decreases the variance across patients. This can most clearly be seen in the least accurate patient LA01, where the mislabeled white matter contacts are less confidently mislabeled.

The dotted line in Figure 6 with slope 1 represents the average performance for a random classifier that, given a threshold τ ∈ [0, 1], labels a contact as white matter with probability τ. For reference, the deterministic classifier that guesses gray matter for all contacts is the same as the random classifier with a threshold of 0, and would be at the point (0, 0) on the graph. This demonstrates why we use ROC curves rather than a simple percentage correct out of the total number of contacts. We might think that if only about 30% of contacts are in white matter, a “dumb” classifier that labels everything as gray matter should have 70% accuracy. In fact it does, but yet it fails as a useful classifier because it has no ability to detect true positives, and its position at (0, 0) on the ROC graph reflects this.

In four-fold cross validation, the training sets are reduced by 25% compared to leave-one-out, resulting in a slightly decreased average AUC of 0.790±0.115 (SD) when integrating over the posterior distribution of β. The low dimensionality of our method and integration over parameters helps to minimize the drop in performance, although we can see that having more training data is generally beneficial.

**Figure 7:**
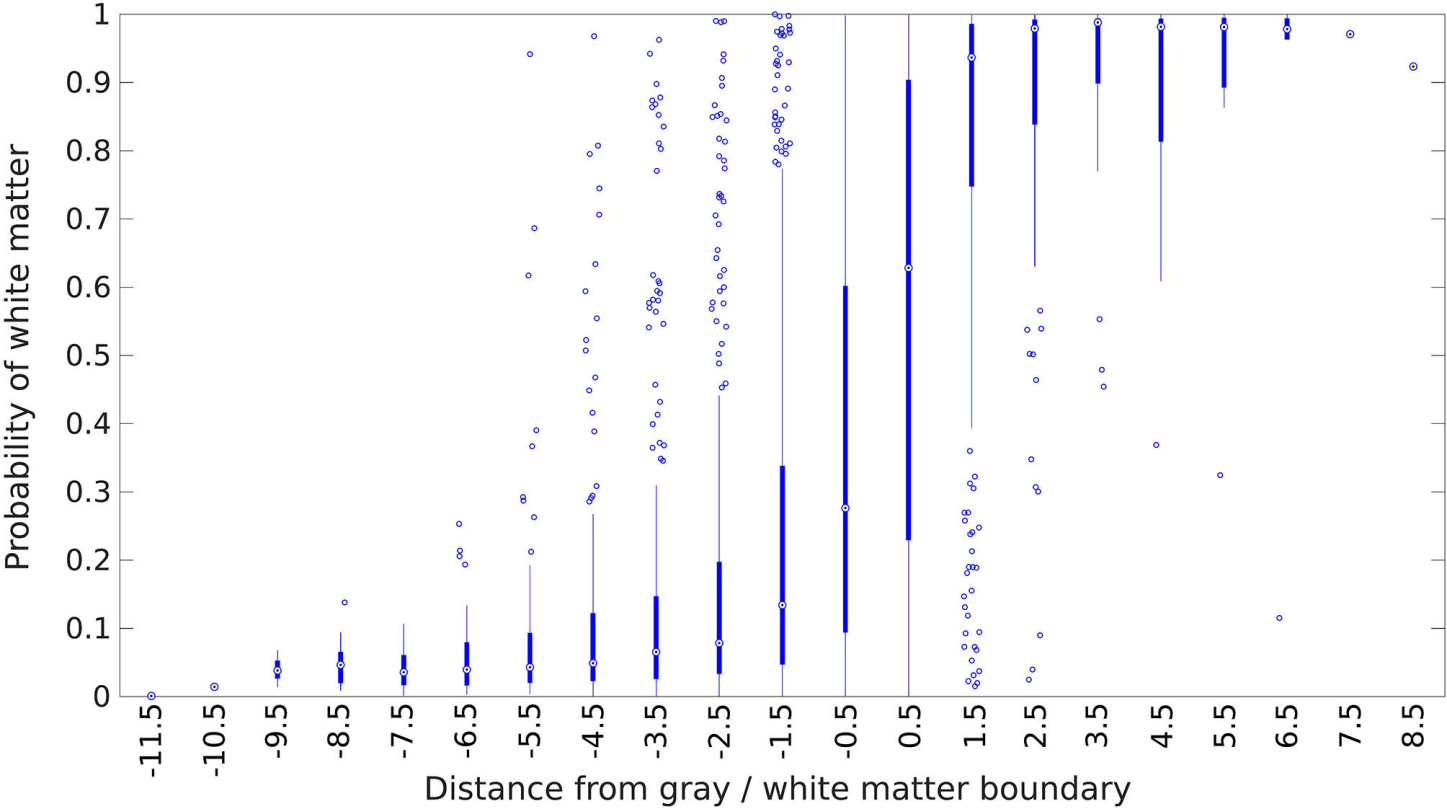
Estimated probability of white matter for electrode contacts as a function of distance from the nearest white matter/gray matter transition. The transition point is defined to be at *x* = 0, and the first contact on the white matter side is at *x* = 0.5, the second contact is at *x* = 1.5, and so forth in unit increments. Positive distances indicate that the contact is deeper into a white matter region, while negative distances indicate the same for gray matter regions. The center circle of each box plot is the median, the thick line indicates the first and third quartiles, and the thin line represents an additional 1.5 times the interquartile range. Points outside this are plotted individually as circles.

Given the fact that the electrical signals produced by gray matter regions propagate some distance into neighboring white matter rather than stopping abruptly at the boundary, we would expect white matter contacts to have signals that are more similar to gray matter the closer they are to the boundary. Our classifier should therefore be less confident (i.e., assign probabilities closer to 0.5) for contacts near a gray/white matter boundary, and more confident for contacts that are deeper in white matter. A similar argument applies to gray matter contacts, as those close to a white matter region receive less signal from the white matter direction, and thus should have lower signal power overall. One place where we know gray/white matter boundaries occur is where there are transitions in the electrode labeling from gray to white or vice versa. Figure 7 shows how our classifier's output probabilities change as a function of distance from these transition points. As expected, contacts near the transition between a gray matter and white matter region tend to be more uncertain, as shown by the median value closer to 0.5, and less accurately assigned, as shown by the larger spread in probabilities, while contacts deeper within white or gray matter are more confidently and accurately assigned.

## 4. Discussion

### 4.1. Feature Selection

In preliminary testing, we investigated several potential features, including the difference in white and gray matter power spectra in the common (also called monopolar) reference signal. The common reference signal is taken relative to one common reference used by all contacts (not to be confused with common average referencing). It is often used by clinicians in conjunction with the bipolar reference signal when examining epilepsy patients and thus seems like an obvious feature candidate. While we found that white and gray matter had somewhat different power spectral densities in the common reference signal, this difference was often much less pronounced than in the bipolar signal and varied more depending on the frequency. This can be seen in Figure 2. We believe this is because the bipolar reference signal takes advantage of the fact that signals in white matter are both smaller and more correlated due to their increased distance from neural sources.

Specifically, if we view the signal on each contact as a combination of a shared source detected across neighboring contacts with some amplitude decay from one contact to the next and an independent local source detected on only the nearest contact, then contacts in white matter will tend to be farther from both their shared and local sources (since the sources must be located in gray matter). If the signal from these sources decays inversely with distance, then the local contributions in white matter contacts will tend to be smaller, and the shared portion of the signal will be closer in magnitude across neighboring electrodes because the rate of decay in signal strength is slower further from the source. Subtracting the signals on neighboring electrodes therefore tends to reduce the signal strength on white matter contacts relatively more than on gray matter contacts, resulting in a larger contrast between the two. We refer the reader to the Supplementary Material for a more in-depth exposition of this argument. Although we expect this to be the typical situation, there can be scenarios where the bipolar signal on white matter contacts remains large due to the geometry of the sources. For example, two contacts within a narrow channel of white matter may have sizable local contributions from separate patches of adjacent gray matter, resulting in a bipolar signal that remains relatively large.

In practice we found that out of 29 patients analyzed, 19 had better AUC when using bipolar referencing, while the remaining 10 patients had better AUC under common referencing. For those patients who did better under common referencing, the difference was often slight. As a result, we did not find an improvement in average AUC when using both common and bipolar referenced features, as opposed to using only bipolar referenced features.

Our second feature, the contact depth, was less important than the first feature, increasing AUC by about 3 percentage points over using the first feature alone. However, it still contributed positively to overall accuracy by encoding the coarse spatial distribution of white and gray matter along an electrode. When an electrode is inserted, it must first pass through the cortex before entering white matter, resulting in the outermost electrodes almost always being in gray matter. The next set of electrodes toward the middle are then more likely to be in white matter, although there can be substantial variation depending on the brain region and angle of insertion due to the anatomy of gyri and sulci. The contacts at the end of an electrode are often used to record from deeper brain areas, and thus are more likely to be in gray matter again. These general tendencies are reflected in the distribution shown in Figure 4.

### 4.2. Uncertainty Quantification

One significant aspect of our classifier is that, rather than giving a single labeling for each electrode, as would be the case in a maximum likelihood classifier or support vector machine, it gives a marginal probability for each contact, and this probability is computed with respect to a flexible and explicit distribution (the kernel density estimate), unlike for example logistic regression where probabilities can be computed but the underlying distribution is implicit and restricted to the exponential family. Furthermore the probabilities take into account uncertainty in the parameter estimates through the posterior predictive calculation, which none of the above methods do and which can be important for small training sets.

For clinical use, we consider giving accurate uncertainty estimates nearly as important as giving the right answer. Any classification method will result in errors, but when the uncertainty in a labeling is given accurately, it allows the clinician to ignore a large fraction of those. Defining the “confidence” that our classifier has in a labeling by 2|*p* − 0.5|, where *p* is the estimated probability of white matter, we find that on mislabelings (defined as *p* > 0.5 for a gray matter contact or *p* < 0.5 for a white matter contact), our classifier had an average confidence level of 54.7, vs. 76.6% for correct labelings. Giving useful probabilities means accurately estimating the underlying data distributions, which becomes increasingly difficult as the number of features increases. This is related to overfitting and can occur even when test set accuracy is very good. Neural networks for example, will typically use hundreds of features and obtain good accuracy, but at the cost of output layer “probabilities” that may not correspond to real error rates or can vary wildly with small perturbations of the input.

### 4.3. Interpretability

Interpretability of features is another aspect we feel is relevant for clinical usefulness. Because of our decision to use features that are explicitly related to the end goal of selecting a subset of informative channels for seizure localization, our classifier's mistakes tend to be clinically acceptable. A white matter contact that has an above-average signal power has a higher chance of being mislabeled as gray matter, but the fact that it is capturing a strong signal means that clinicians may want to keep this channel anyway as it is likely to be picking up signal from an adjacent gray matter region. It is also possible that there are errors in the clinician-labeled ground truth classifications, and that in some cases it may be the clinician labeling which is wrong rather than our classifier. Several patients had files which indicated uncertainty about some of the labeled white matter contacts, showing that even with MRI and CT, it can be difficult to determine whether some contacts are in white or gray matter, and that a signal-based classifier may be useful as an independent reference.

### 4.4. Computational Efficiency

Our classifier takes less than 5 min to run per patient, much faster than the typical hand-labeling process using MRI and CT which can take several hours. In theory, the evaluation of the likelihood in the posterior predictive requires a sum over a number of terms that grows quadratically with the size of the number of contacts in the data set, due to the use of a gaussian kernel estimator to evaluate the distribution rather than a parametric model. For large datasets, one can reduce this somewhat by using a kernel with finite support rather than a gaussian. As the number of data points increases, the estimated kernel width will become narrower, reducing the growth in the number of points within the support of the kernel. For very large datasets we recommend simply using a subset as the training data; since our model is low-dimensional, it is not necessary to use vast amounts of data to estimate the feature distributions, and using a random training subset of a few dozen patients will likely yield nearly identical results.

The run time of our classifier can also be adjusted by changing the number of samples drawn from the posterior distribution of the parameters when computing the posterior predictive. With our truncated gaussian approximation, drawing samples is fast but evaluating the posterior probability of a labeling is slow due to having to recalculate the normalizing constant for each sample. In our case, we have sufficient training data that our posterior parameter distributions are tightly peaked, and thus relatively few samples are needed. The required number of samples depends on the desired amount of variance in the posterior predictive estimate. If one has less than 20 or so patients, more extensive sampling will likely be needed, with total computation time increasing linearly with the number of samples.

### 4.5. Future Work

One limitation of our current approach is the way in which we compute the distribution of our second feature, the location of white or gray matter along the electrode. For simplicity we pool the data from all electrodes, giving us a coarse average distribution which ignores the differences between electrodes inserted in different brain regions or at different angles. An improvement on this would be to estimate separate distributions as functions of the brain region and other implantation variables, which would allow more detailed structure to be captured and improve the usefulness of this feature. Further testing would be needed to explore the trade-off between accuracy and the amount of extra data needed to estimate the distributions.

Additional refinements to our method are possible, for example the sample-based estimation of the posterior predictive could be parallelized to further increase speed. Another avenue for improvement would be the use of common reference data in addition to bipolar reference data. As described above, although the majority of contacts are better distinguished using bipolar data, there are undoubtedly a fraction of contacts which can be more accurately classified using the common reference. Simply adding the common reference power as a third feature does not improve accuracy by a useful amount however. What is needed is an additional feature that determines whether a contact is more likely to benefit from using the bipolar or common reference. The previous discussion (also see Supplementary Material) suggests some possibilities which we leave for future work, such as estimating the fraction of common vs. local variance.

## Data Availability Statement

The data analyzed in this study is subject to the following licenses/restrictions: Patient data to be used for approved research/medical purposes only. Requests to access these datasets should be directed to Jorge González-Martínez, jalmartinez@sbcglobal.net.

## Ethics Statement

The studies involving human participants were reviewed and approved by the Cleveland Clinic Institutional Review Board. The patients/participants provided their written informed consent to participate in this study.

## Author Contributions

PG developed the method with support from SS and implemented it. PG wrote the manuscript with contributions from AL, SS, and JG-M. JG-M provided clinical information and raw patient data. AL organized the data into the BIDS format. All authors contributed to the article and approved the submitted version.

## Conflict of Interest

The authors declare that the research was conducted in the absence of any commercial or financial relationships that could be construed as a potential conflict of interest.
