# Supplementary material for: Classification of Stereo-EEG Contacts in White Matter vs. Gray Matter Using Recorded Activity
Source: Front Neurol. 2021 Jan 6;11:605696. doi: 10.3389/fneur.2020.605696 (PMC7815703; doi:10.3389/fneur.2020.605696)
Supplement: Supplementary file 1 [file Data_Sheet_1.PDF]

Supplementary Material

1 MATHEMATICAL DESCRIPTION OF CLASSIFIER

For contact $i = 1, \ldots, N_s$ on electrode shank s with observed feature values $\boldsymbol{x}_{s,i} = (x_{s,i,1}, x_{s,i,2})$ of spectral power difference and contact depth, we wish to determine the corresponding labeling $z_{s,i}$, where $z_{s,i} \in \{-1, 1\}$, with -1 denoting gray matter and 1 denoting white matter. Assume that we have some labeled training data $D = \{\boldsymbol{z}_t\}_T, \{\boldsymbol{x}_t\}_T$ with T indexing the training set. From the graphical model, which represents the test set (where D is omitted for clarity, but is observed and connects to all the other nodes), we see that the joint distribution of $\boldsymbol{x}_s, \boldsymbol{z}_s, \boldsymbol{\alpha}, \beta, D$ (where $\boldsymbol{x}_s, \boldsymbol{z}_s$ are the data and labels for shank s in the test set and $\boldsymbol{\alpha}$, β are parameters) can be written as a product of functions of the form:

$$p(\boldsymbol{x}_s, \boldsymbol{z}_s, \boldsymbol{\alpha}, \beta, D) = C \prod_{i=1}^{N_s} f_i(z_{s,i}, \boldsymbol{x}_{s,i}, \boldsymbol{\alpha}, D) g_i(z_{s,i}, z_{s,i+1}, \beta, D) \tag{S1}$$

where C is a normalizing constant. For $i = 1, \ldots, N_s - 1$, we take f_i to be the probability of $\boldsymbol{x}_{s,i}$, and g_i to be the prior probability of $z_{s,i}, z_{s,i+1}$. For $i = N_s$, we further multiply f_i by the posterior distribution of $\boldsymbol{\alpha}$ and set g_i to the posterior distribution of β:

$$f_i = \begin{cases} p(\boldsymbol{x}_{s,i} | z_{s,i}, \boldsymbol{\alpha}, D) & i = 1, \ldots, N_s - 1 \\ p(\boldsymbol{x}_{s,i} | z_{s,i}, \boldsymbol{\alpha}, D) p(\boldsymbol{\alpha} | D) & i = N_s \end{cases} \tag{S2}$$

$$g_i = \begin{cases} p(z_{s,i}, z_{s,i+1} | \beta) & i = 1, \ldots, N_s - 1 \\ p(\beta | D) & i = N_s \end{cases} \tag{S3}$$

To simplify probability calculations, we assume some stronger independence statements than the graphical model makes explicit. Since the distributions of parameters $\boldsymbol{\alpha}, \beta$ are estimated on the training data (where $\{\boldsymbol{z}_t\}_T$ are observed) and held fixed on the test set, they are independent given the training data D, and their posterior distributions are independent of $\boldsymbol{z}_s$. In addition, $\boldsymbol{z}_s$ is taken to be independent of D and $\boldsymbol{\alpha}$ given β. Under these assumptions, along with the conditional factorization of $\boldsymbol{x}_s$ and $\boldsymbol{z}_s$ shown in the graphical model, we see that as long as each distribution p is a proper probability distribution, the joint distribution defined in this way integrates to $1/p(D)$. Thus if we divide out $C = p(D)$, the product above gives us the joint posterior $p(\boldsymbol{x}_s, \boldsymbol{z}_s, \boldsymbol{\alpha}, \beta | D)$. For reference, if we marginalize out $\boldsymbol{x}_{s,i}$ and $\boldsymbol{\alpha}$ for all i and divide by $p(\beta | D)$, we have the distribution of $\boldsymbol{z}_s$ as the product:

$$p(\boldsymbol{z}_s | \beta) = \prod_i p(z_{s,i}, z_{s,i+1} | \beta) \tag{S4}$$

We now give explicit forms for each of the distributions above. We use kernel density estimators with gaussian kernels $K_{\boldsymbol{\alpha}}$ parametrized by the vector of kernel widths $\boldsymbol{\alpha}_{wm} = (\alpha_{wm,1}, \alpha_{wm,2})$ or $\boldsymbol{\alpha}_{gm} = (\alpha_{gm,1}, \alpha_{gm,2})$ to compute data likelihoods for the white matter and gray matter distributions (denoted by the wm and gm subscripts). We will write the set of kernel width parameters as $\boldsymbol{\alpha}$, where $\boldsymbol{\alpha}_{gm}$ or $\boldsymbol{\alpha}_{wm}$ is

chosen depending on $z_{s,i}$ when computing $p(\boldsymbol{x}_{s,i}|z_{s,i}, D, \boldsymbol{\alpha})$. The distance between contacts is normalized to 1 in our model, so the distance along the shank is really a discrete distribution with mass at the integers. The use of a continuous kernel density to estimate it allows information about the spatial distribution of white and gray matter to be shared across neighboring contacts. Evaluating the probability at a given datapoint $\boldsymbol{x}_{s,i}$ (where $x_{s,i,2} \in \{0, 1, 2...\}$ denotes the contact depth) then requires re-assigning probability mass to the nearest integer, which we do by integrating the continuous distribution along this dimension from $x_{s,i,2} - 0.5$ to $x_{s,i,2} + 0.5$:

$$p(\boldsymbol{x}_{s,i}|z_{s,i}, D, \boldsymbol{\alpha}) = \int_{x_{s,i,2}-0.5}^{x_{s,i,2}+0.5} \frac{1}{|J|} \sum_{j \in J} K_{\boldsymbol{\alpha}}(\boldsymbol{x}'_{s,i,2} - \boldsymbol{x}_j) dx'_{s,i,2} \tag{S5}$$

where J is the subset of the training set corresponding to $z_{s,i}$ (i.e. all white matter or gray matter contacts) and $|J|$ is its size. Separate contacts on an electrode are taken to be conditionally independent given $\boldsymbol{z}, \boldsymbol{\alpha}$ as shown in the graphical model.

Given the physical structure of the brain, we would find it unlikely for an electrode to have contacts that alternate between white matter and gray matter in rapid succession. More typical would be to have several gray matter contacts in a row, followed by several white matter contacts in a row, perhaps followed by another chunk of gray matter contacts. This tendency for neighboring contacts to have the same classification is captured by our prior distribution on labelings:

$$p(\boldsymbol{z}_s|\beta) = \frac{1}{C(\beta, N_s)} \exp(\frac{1}{N_s} \sum_{i=1}^{N_s-1} \beta\, z_{s,i} z_{s,i+1}) \tag{S6}$$

where $C(\beta, N_s)$ is a normalizing constant that depends both on the prior parameter $\beta > 0$ and the number of contacts N_s on the particular electrode. We do not assume that every electrode will have the same number of contacts; due to malfunctions and the depth at which an electrode is inserted, the number of usable contacts will often be different across electrodes within a patient. Large values of β penalize white matter/gray matter transitions more and lead to smoother solutions. The $1/N_s$ in the exponential has been added to account for the different numbers of contacts across electrodes and slightly improves accuracy when using a common β for all electrodes.

By Bayes' rule, the probability of a given vector of labels $\boldsymbol{z}$ for all contacts on electrode s is given by:

$$p(\boldsymbol{z}_s|\boldsymbol{x}_s, D, \boldsymbol{\alpha}, \beta,) = \frac{\prod_i p(\boldsymbol{x}_{s,i}|z_{s,i}, D, \boldsymbol{\alpha}) p(\boldsymbol{z}_s|\beta)}{p(\boldsymbol{x}_s|D, \boldsymbol{\alpha}, \beta)} \tag{S7}$$

where we have conditioned on the parameters $\boldsymbol{\alpha}, \beta$. The denominator is computed by summing the numerator over all possible labelings $\boldsymbol{z}_s$. This has 2^{N_s} terms, but can be computed in a few seconds for any electrode with N_s less than about 20 (which includes all electrodes in our dataset) by first computing $p(\boldsymbol{x}_{s,i}|z_{s,i} = 1, D, \boldsymbol{\alpha})$ and $p(\boldsymbol{x}_{s,i}|z_{s,i} = -1, D, \boldsymbol{\alpha})$ for all i and storing the values, then multiplying these together as appropriate for the given $\boldsymbol{z}_s$.

We allow the user to explicitly choose the kernel width $\boldsymbol{\alpha}_2 = (\alpha_{2,wm}, \alpha_{2,gm})$ in the second feature dimension corresponding to the contact depth, as described in the Methods section.

The kernel width $\boldsymbol{\alpha}_1 = (\alpha_{wm,1}, \alpha_{gm,1})$ in the first feature dimension (spectral power difference) and the prior smoothing parameter β lack an intuitive physical length scale to guide their selection, and thus we deal with these automatically. Rather than choosing one particular value for these parameters, we prefer to integrate them out with respect to their posterior distribution $p(\boldsymbol{\alpha}_1, \beta|D)$, forming the posterior predictive:

$$p(\boldsymbol{z}_s|\boldsymbol{x}_s, D, \hat{\alpha}_2) = \int p(\boldsymbol{z}_s|\boldsymbol{x}_s, D, \boldsymbol{\alpha}, \beta) p(\boldsymbol{\alpha}_1, \beta|D) d\boldsymbol{\alpha}_1 d\beta \tag{S8}$$

where $\hat{\alpha}_2$ is the fixed value of α_2 as set by the user. This takes into account uncertainty in the values of the parameters and, especially for smaller training sets where $\boldsymbol{\alpha}$ and β are not sharply estimated, gives posterior probabilities of $\boldsymbol{z}$ that more accurately reflect the true uncertainty in classification.

The first term is calculated according to equation S7. Since each electrode shank is independent, the posterior distribution of the parameters themselves is given by:

$$p(\boldsymbol{\alpha}_1, \beta|D) = \frac{p(\{\boldsymbol{x}_t\}_T|\{\boldsymbol{z}_t\}_T, \boldsymbol{\alpha}_1, \beta) p(\boldsymbol{\alpha}_1, \beta|\{\boldsymbol{z}_t\}_T)}{p(\{\boldsymbol{x}_t\}_T|\{\boldsymbol{z}_t\}_T)} \tag{S9}$$

$$= \frac{p(\boldsymbol{\alpha}_1) \prod_{t \in T} p(\boldsymbol{x}_t|\boldsymbol{z}_t, \boldsymbol{\alpha}_1)}{p(\{\boldsymbol{x}_t\}_T|\{\boldsymbol{z}_t\}_T)} \cdot \frac{\prod_{t \in T} p(\boldsymbol{z}_t|\beta) p(\beta)}{p(\{\boldsymbol{z}_t\}_T)} \tag{S10}$$

where again $\boldsymbol{x}_t, \boldsymbol{z}_t$ are the data for electrode shank t in the training set T. We use the fact that $\boldsymbol{\alpha}_1$ and β are independent given $\{\boldsymbol{z}_t\}$ (on the training set). The prior over $\boldsymbol{\alpha}_1$ has components $\alpha_{wm,1}, \alpha_{gm,1}$ which are assumed independent with exponential distributions since we require $\alpha > 0$:

$$p(\alpha_{wm,1}) = \lambda e^{-\lambda \alpha_{wm,1}} \tag{S11}$$

and similarly for $\alpha_{gm,1}$. We set $\lambda = 0.1$ to make the prior relatively diffuse; its selection thus had negligible effect on final estimated probabilities.

Because our data model is defined via a kernel density estimate which directly uses the training data to evaluate new points (note the D in equation S5), it is unclear how to evaluate the likelihood term $p(\boldsymbol{x}_t|\boldsymbol{z}_t, \boldsymbol{\alpha}_1)$ for a point in the training set itself. Conditioning on the training set again is unhelpful in that we would be evaluating $p(\boldsymbol{x}_t|\boldsymbol{x}_t)$; even ignoring that, the likelihood would diverge to infinity as the kernel width approached 0. One solution is to consider the likelihood in this case as an expected likelihood over all samples $\tilde{D}$ drawn from the true data distribution $p(D)$:

$$p(\boldsymbol{x}_t|\boldsymbol{z}_t, \boldsymbol{\alpha}_1) = \int p(\boldsymbol{x}_t|\boldsymbol{z}_t, \tilde{D}, \boldsymbol{\alpha}_1) p(\tilde{D}) d\tilde{D} \tag{S12}$$

We do not know the true data distribution, but for $\boldsymbol{\alpha}$ that allows the data probability to be accurately represented at all points, the value of the likelihood should not change much depending on the particular sample $\tilde{D}$ we observe, nor should it change much if the size of T is slightly different. This suggests approximating the average by the value at the single sample we do have (the training set), minus the point we are evaluating:

$$p(\boldsymbol{x}_t|\boldsymbol{z}_t, \boldsymbol{\alpha}_1) \approx p(\boldsymbol{x}_t|\boldsymbol{z}_t, D_{-t}, \boldsymbol{\alpha}_1) \tag{S13}$$

where D_{-t} is the training set with $\boldsymbol{x}_t$ removed. Although this allows us to evaluate $p(\boldsymbol{\alpha}_1, \beta|D)$, it is a fairly expensive computation, requiring a sum over $O(|T|^2)$ terms due to the sum in equation S5. We wish to avoid having to evaluate it the hundred or more times necessary for calculating the three-dimensional integral in equation S8, since the first term is itself also slow to compute, and these combined would make our classifier prohibitively slow. We thus find its peak and approximate it by a truncated gaussian centered at this point (since neither $\boldsymbol{\alpha}$ nor β can be negative), then estimate the integral by averaging the first term over samples drawn from this gaussian. In the results shown, we average across 100 samples.

Finally, we wish to compute the probability that $z_{s,i} = 1$ (white matter) for each contact individually. We do this by marginalizing out the labels of all the other contacts in equation S8. This comes at almost no additional cost if we save the terms in the sum over $\boldsymbol{z}_s$ needed to determine the denominator of equation S7, because for each value of $\boldsymbol{\alpha}_1, \beta$ the marginal is simply a partial sum over the terms with $z_{s,i} = 1$.

2 BIPOLAR VERSUS COMMON REFERENCING

To see why bipolar referencing tends to work better than common (monopolar) referencing for distinguishing between contacts in white and gray matter, consider the following simplified model of the voltage time series on contact X in gray matter and contact Y on the same shank but in white matter. Ignoring the temporal dependence of observed voltages which gives the particular shape of the power spectrum, we recall that the primary difference between the white and gray matter spectrums was closely related to the difference in total power (integral of the PSD over all frequencies), with white matter contacts having lower total power. If we consider this signal as a stochastic process with independent and identically distributed (i.i.d.) random variables, the total power is proportional to their variance. In this light, we model the time series for contact X by a sequence of i.i.d. random variables $\{X_t\}$ indexed by time t, and similarly for Y. Assume that we have removed any residual DC component so that the mean signal for any contact is 0 ($\mathbb{E}(X_t) = 0$ for all t and same for Y). The smaller average signal amplitude seen on white matter contacts at a given time can be quantified as the ratio of the variances (var). In a common reference (CR) signal, this would be:

$$\text{CR amplitude ratio} \equiv \frac{var(X_t)}{var(Y_t)} \tag{S14}$$

The larger this amplitude ratio, the more clearly we can distinguish contacts in white matter from gray matter. Let X', Y' be the neighboring contacts to X and Y, respectively. The amplitude ratio in a bipolar (BP) reference signal would then be:

$$\text{BP amplitude ratio} \equiv \frac{var(X_t - X'_t)}{var(Y_t - Y'_t)} \tag{S15}$$

$$= \frac{var(X_t) + var(X'_t) - 2\,cov(X_t, X'_t)}{var(Y_t) + var(Y'_t) - 2\,cov(Y_t, Y'_t)} \tag{S16}$$

where $cov()$ is the covariance. If the signals on neighboring contacts are independent and have the same variance, we would get the same amplitude ratio as in common referencing, and there would be no

advantage to using bipolar referencing in this case. However, consider the more realistic case where the signal on $X'$is an attenuated version of a common signal C_t shared between X and X', plus an independent, local signal detected only at X' (and similarly for Y and Y', where we denote the common signal D_t):

$$X_t = C_t + \epsilon_t \tag{S17}$$
$$X'_t = \alpha_X C_t + \epsilon'_t \tag{S18}$$
$$Y_t = D_t + \gamma_t \tag{S19}$$
$$Y'_t = \alpha_Y D_t + \gamma'_t \tag{S20}$$

where $0 < \alpha_X, \alpha_Y \leq 1$ are the attenuation factors and $\epsilon_t, \epsilon'_t, \gamma_t, \gamma'_t$ are the independent local signals. If the source were closer to X' than X we would just do the analysis in terms of X' rather than X, so the $\alpha \leq 1$ assumption does not affect generality. Under this model, the CR amplitude ratio is given by:

$$\frac{var(C_t) + var(\epsilon_t)}{var(D_t) + var(\gamma_t)} \tag{S21}$$

and the BP amplitude ratio is:

$$\frac{(\alpha_X - 1)^2 var(C_t) + var(\epsilon_t) + var(\epsilon'_t)}{(\alpha_Y - 1)^2 var(D_t) + var(\gamma_t) + var(\gamma'_t)} \tag{S22}$$

Assuming that the signals on neighboring electrodes have the same variance, we can rewrite $var(\epsilon'_t)$ in terms of the other variances:

$$var(X_t) = var(X'_t) \tag{S23}$$
$$\implies \quad var(\epsilon'_t) = (1 - \alpha_X^2) var(C_t) + var(\epsilon_t) \tag{S24}$$

and similarly for $var(\gamma'_t)$. This implies X', being further from the common source, has proportionally more of its signal coming from the local source. Substituting equation S24 into S22, we obtain for the BP amplitude ratio:

$$\frac{(1 - \alpha_X) var(C_t) + var(\epsilon_t)}{(1 - \alpha_Y) var(D_t) + var(\gamma_t)} \tag{S25}$$

Note that this is the CR amplitude ratio with $-\alpha_X var(C_t)$ added to the numerator and $-\alpha_Y var(D_t)$ added to the denominator. After some algebra, we find that BP amplitude ratio $>$ CR amplitude ratio when:

$$\frac{\alpha_X}{\alpha_Y} < \frac{var(C_t) + var(\epsilon_t)}{var(C_t) + \delta\, var(\epsilon_t)} \tag{S26}$$

$$\delta \equiv \frac{var(C_t)}{var(D_t)} \frac{var(\gamma_t)}{var(\epsilon_t)} \tag{S27}$$

Before discussing equation S26, we first remark on the attenuation ratio of the common sources α_X/α_Y. Suppose that the source potential decays approximately as $f(r) = k/r^p$ for $p > 0$, where r is the distance from the source and k is some constant. $p = 1$ represents a monopole source, $p = 2$ a dipole source (often assumed in seizure localization), and so forth. The amplitude attenuation factor between two contacts at points r_1, $r_1 + \Delta r$ is the number α_r such that:

$$f(r_1 + \Delta r) = \alpha_r f(r_1) \tag{S28}$$

$$\implies \quad \alpha_r = \frac{f(r_1 + \Delta r)}{f(r_1)} \tag{S29}$$

$$= \left(\frac{r_1}{r_1 + \Delta r}\right)^p \tag{S30}$$

We can see that $0 < \alpha_r \leq 1$, and $\alpha_r \to 0$ as $r_1 \to 0$ (the contact gets closer to the source), and $\alpha_r \to 1$ as $r_1 \to \infty$ (the contact gets further from the source). Typically we would expect that contacts in gray matter would be closer to their common source than contacts in white matter, so that $\alpha_X < \alpha_Y$, and thus

$$\frac{\alpha_X}{\alpha_Y} < 1 \tag{S31}$$

Returning to equation S26, we now wish to estimate how large the right side is. Assuming the proportion of variance from the common source relative to the local source is about the same in white matter as it is in gray matter, δ should be close to 1:

$$\frac{var(\gamma_t)}{var(D_t)} \approx \frac{var(\epsilon_t)}{var(C_t)} \tag{S32}$$

$$\implies \quad \delta \approx 1 \tag{S33}$$

By assumption, the common source is detected across multiple contacts. This can happen if the common source decays more slowly with distance than the local source due to its spatial arrangement. For example, if it is spread out over a larger region of tissue, or the currents are otherwise aligned in a way as to produce a lower order multipole, the resulting potential field will have slower spatial decay. In this case we would typically expect the common source to contribute a larger fraction of the total variance in white matter because both sources are further away from the contact. If r is the distance from the common source to a contact, and the local source is a distance $r - \hat{r}$ from the contact for a fixed $\hat{r}$ with $0 \leq \hat{r} < r$ (i.e. the

local source is assumed to be the same distance or closer to the contact than the common source), and the common and local sources decay at rates p_1, p_2 respectively, with $0 < p_1 \leq p_2$, then:

$$\frac{\text{common source strength}}{\text{local source strength}} = \frac{k_1\,(1/r)^{p_1}}{k_2\,(1/(r-\hat{r}))^{p_2}} \tag{S34}$$

$$= \frac{k_1}{k_2}\left(\frac{r-\hat{r}}{r}\right)^{p_2} r^{p_2-p_1} \tag{S35}$$

where k_1, k_2 are positive constants. Ignoring the constants, both terms in equation S35 increase monotonically with r, with the first term approaching 1 and the second term going to infinity. We expect r to be larger in white matter than in gray matter, hence equation S35 shows that the common source ought to contribute proportionately more to the overall signal in this case. If the common and local sources have the same decay rate but the common source is simply stronger (i.e. the local source falls below the detection level at the neighboring contact), then we still have the same conclusion, although the effect would be weaker. To see this, set $p_1 = p_2$ and note that while the third term is just 1, the second term remains constant (if $\hat{r} = 0$) or monotonically increasing with r, though bounded. In any of the above scenarios, which we believe represent typical scenarios for the relationship between the local and common sources, we would have a larger proportion of the variance coming from the common source in white matter than in gray matter: $var(\gamma_t)/var(D_t) \leq var(\epsilon_t)/var(C_t)$, and hence $\delta \leq 1$. In equation S26, we then have

$$\frac{var(C_t) + var(\epsilon_t)}{var(C_t) + \delta\, var(\epsilon_t)} \geq 1 \tag{S36}$$

as a "typical" situation. Even in cases where $\delta > 1$, the fact that we usually see strong correlation between neighboring contacts suggests $var(C_t) \gg var(\epsilon_t)$, so that the right hand side of S26 is probably never much less than 1.

Combining equation S36 with equation S31, we can conclude that equation S26 is likely to hold for most sets of white and gray matter contacts, meaning that bipolar referencing will often work better than common referencing at distinguishing white from gray matter, which is in line with what we observe when testing our classifier.
